# Interrupted CAG expansions in *ATXN2* gene expand the genetic spectrum of frontotemporal dementias

**DOI:** 10.1186/s40478-018-0547-8

**Published:** 2018-05-30

**Authors:** Clémence Fournier, Vincent Anquetil, Agnès Camuzat, Sandrine Stirati-Buron, Véronique Sazdovitch, Laura Molina-Porcel, Sabrina Turbant, Daisy Rinaldi, Raquel Sánchez-Valle, Mathieu Barbier, Morwena Latouche, Neuro-CEB Neuropathology Network, Franck Letournel, Anne Vital, Françoise Chapon, Catherine Godfraind, Claude-Alain Maurage, Vincent Deramecourt, David Meyronnet, Nathalie Streichenberger, André Maues de Paula, Valérie Rigau, Fanny Vandenbos-Burel, Danielle Seilhean, Serge Milin, Dan Christian Chiforeanu, Annie Laquerrière, Béatrice Lannes, Giovanni Stevanin, Danielle Seilhean, Alexis Brice, Charles Duyckaerts, Isabelle Le Ber

**Affiliations:** 1 Institut du Cerveau et la Moelle épinière (ICM), Sorbonne Université, UPMC Univ Paris 06, Inserm U1127, CNRS UMR 7225, Hôpital Pitié-Salpêtrière, Paris, France; 2 Ecole Pratique des Hautes Etudes – EPHE, PSL research University, 75014 Paris, France; 3 Département de médecine gériatrique, Centre hospitalier Rives de Seine, Courbevoie, France; 4 Neurological Tissue Bank of the Biobanc-Hospital Clinic-IDIBAPS, Barcelona, Spain; 5 Laboratoire de Neuropathologie Escourolle, AP-HP - Hôpital Pitié-Salpêtrière, Paris, France; 6 Department of Neurology, AP-HP - Hopital Pitié-Salpêtrière, Reference center for rare or early dementias, Institute of Memory and Alzheimer’s Disease (IM2A), Paris, France; 7 Alzheimer disease and other Cognitive Disorders Unit, Department of Neurology, Hospital Clinic, Barcelona, Spain; 8 Reference center for neurogenetics, Departement of Genetics, APHP, Hôpital Pitié-Salpêtrière, 75013 Paris, France

**Keywords:** Frontotemporal dementia, Frontotemporal lobar degeneration, TDP-43, Ataxin 2, Amyotrophic lateral sclerosis, C9orf72, SCA2, GRN, Corticobasal degeneration, Corticobasal syndrome

Spinocerebellar ataxia type 2 (SCA2) is due to a CAG repeat expansion in *Ataxin-2* gene (*ATXN2*), encoding a polyglutamine (polyQ) stretch. Thirty-four or more uninterrupted (pure) CAG repeats are associated with cerebellar ataxia, slow saccades, and parkinsonism, beginning before 60 years [1]. SCA2 is associated with neuronal loss in the cerebellum, *substantia nigra*, striatum and globus pallidus; and intranuclear aggregation of polyglutamine stretches, labelled by 1C2 antibody, in the cerebellum [2]. When interrupted by CAA motifs, full CAG expansions produce isolated levodopa-responsive parkinsonism [3]. On the other hand, intermediate alleles greater than 26 [4], and up to 39 CAG repeats [5], represent a strong risk factor for amyotrophic lateral sclerosis (ALS) associated with neuronal TDP-43 (TAR DNA binding Protein 43) inclusions.

*ATXN2* contribution to TDP-43-proteinopathies has been studied mostly in ALS. We evaluated the contribution of *ATXN2* in 31 patients with frontotemporal lobar degeneration (FTLD) and pathologically proven TDP-43 inclusions (FTLD-TDP) without known related mutation (Supplementary methods, Additional file 1: Table S1). One patient (patient 5, Additional file 1: Table S1) carried a 39 CAG expansion interrupted by four CAA motifs (CAG_8_–CAA–CAG_4_–CAA–CAG_4_-CAA-CAG_9_-CAA-CAG_10_), and a 27 CAG intermediate allele (CAG_8_–CAA–CAG_4_–CAA–CAG_4_-CAA-CAG_8_) (Supplementary results). The patient developed agrammatism, word omissions and dysarthria, suggestive of nonfluent primary progressive aphasia, at age 70 (Supplementary results). Brain imaging revealed frontal, left peri-sylvian and parietal atrophy; cerebellum was normal (Fig. 1 a-d). At age 73, the association of marked frontal executive dysfunction (planning, attention, inhibition, mental flexibility deficits), ideomotor apraxia (praxis score: 4/23), akinetic-rigid parkinsonism, with asymmetric fronto-temporo-parietal atrophy, was consistent with a frontal-behavioral subtype of corticobasal syndrome (CBS) [6]. He had no cerebellar syndrome. He died at age 77. No information about the patient’s family was available.

**Fig. 1.**
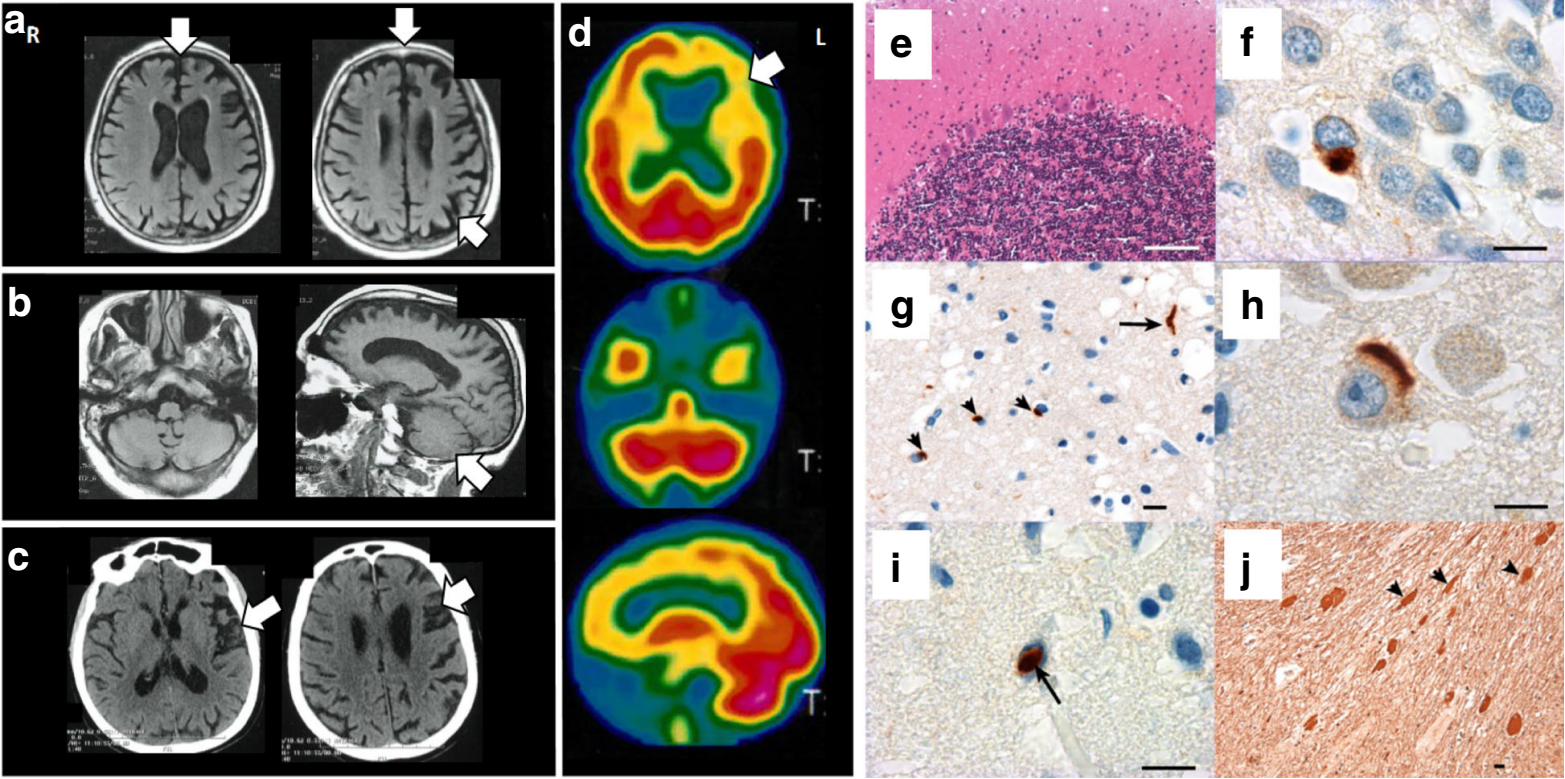
Brain imaging and pathology. Left: Brain MRI and CT scan and HMPAO-SPECT examination of patient 5 (aged 73 years). **a**. Brain T1 axial sections showing marked bilateral frontal atrophy, associated with predominantly left parietal atrophy (arrows). **b**. T1 axial and coronal sections, showing no cerebellar atrophy. **c**. brain CT scan (axial sections) showing predominantly left peri-sylvian and frontal atrophy (arrows). **d**. HMPAO-SPECT examination (axial and coronal sections) showing bilateral, predominantly left (arrow), hypoperfusion. L: left; R: right. Right: Brain pathological lesions of patient 5. **e**. Cerebellum. Haematoxylin-Eosin stain. Normal density of Purkinje cells, of granule cells and of glomeruli. **f**. Dentate gyrus. Phospho-TDP-43 immunohistochemistry. Neuronal cytoplasmic inclusion. **g**. Upper layers of the middle frontal gyrus. Phospho-TDP-43 immunohistochemistry. Several cytoplasmic inclusions (short arrows) in glial cells. One phospho-TDP-43 positive neurite (long arrow). **h**. Middle frontal gyrus. Phospho-TDP-43immunohistochemistry. Neuronal cytoplasmic inclusion. **i**. Middle frontal gyrus. Phospho-TDP-43 immunohistochemistry. Cat-eye nuclear inclusion (arrow). **j**. Medulla oblongata. Neurofilamentimmunohistochemistry. Numerous axonal spheroids (arrows) in the amiculum of the inferior olives. All scale bars= 10 μm

A post-mortem examination was performed (Supplementary methods and results). Both 39 and 27 CAG alleles were found in all studied brain structures (frontal cortex, striatum, mesencephalon, occipital cortex, cerebellum) (Additional file 1: Figure S1 and Figure S2). Macroscopic examination revealed marked atrophy of frontal, temporal lobes, Ammon’s horn (CA1) and the subiculum. Neuronal loss and gliosis, associated with a superficial laminar spongiosis, were severe in the superficial layers of the middle frontal gyrus, motor cortex, supramarginal gyrus, CA1 and the subiculum. The pons (including the *locus coeruleus*), the cerebellum (Fig. 1e) and the dentate nucleus were normal. TDP-43, pTDP-43, p62 and ubiquitin immunohistochemistry revealed small round cytoplasmic inclusions, sometimes glial, more abundant in the superficial layers of the middle frontal gyrus, motor cortex, and supramarginal gyrus (Fig. 1e-j). Rare pTDP-43 ‘cat eye’ intranuclear inclusions were detected (Fig. 1i). The presence of TDP-43 positive cytoplasmic inclusions, mainly distributed in the upper layers of the cortex, lead to the diagnosis of type A FTLD-TDP [7]. Few cytoplasmic inclusions were found in the dentate gyrus (Fig. 1f). Scarce TDP-43 and pTDP43 positive neurites were present, mainly in the frontal cortex and the supramarginal gyrus (Fig. 1g-i). No skein like inclusions were observed in the hypoglossal nucleus. Ubiquitin and p62 immunohistochemistry did not reveal inclusion in the cerebellum. No intranuclear inclusions were detected with 1C2 antibodies. No alpha-synuclein immunostaining was noted in the *substantia nigra*. Ataxin2 immunochemistry revealed granular cytoplasmic staining in Purkinje cells of the cerebellum and in neurons of spinal cord, similar to that found in an ALS case with intermediate 32 CAG expansion, and weak diffuse cytoplasmic staining in some neurons of the frontal cortex (Additional file 1: Figure S3).

The presentation by CBS, without cerebellar ataxia, or cerebellum lesions on imaging and pathological examination (Fig. 1b, e), was clearly distinct from SCA2 phenotype. The neuropathology, characterized by pTDP-43-positive inclusions in the neocortex, but no cerebellar lesions or 1C2 inclusions, was also different from SCA2 patients (Additional file 1: Table S2). The phenotype was distinct from the late levodopa-responsive parkinsonism associated with interrupted expansions [3, 8]. Lastly, Lewy bodies and 1C2-positive inclusions in *substantia nigra*, pontine nuclei and cerebellum, described in few parkinsonian patients [9], were absent in our case. This study shows that *ATXN2* phenotypes are not restricted to cerebellar ataxia, parkinsonism and ALS, but are expanded to pure isolated FTLD phenotypes. Although a coincidental occurrence of FTLD-TDP and *ATXN2* mutation cannot be formally excluded, all known FTLD and ALS genes were normal in our patient. More importantly, an interrupted *ATXN2* expansion was previously identified in a patient with FTD-ALS and type B FTLD-TDP pathology [10], both cases thus strongly support the causative genetic link between FTLD-TDP and *ATXN2*.

The clinical variability of *ATXN2* expansions is not fully explained. In most repeat expansion disorders, somatic mosaicism of the expanded alleles contributes to the clinical expression. Conversely to pure *ATXN2* expansions, no (or a low level of) brain mosaicism was observed in this case, probably because interrupted expansions are more stable than pure ones. Interrupted expansions are also more stable than uninterrupted ones across meiosis [3]. It possibly confers a risk of anticipation lower than in pure CAG repeats, which should be considered in genetic counselling. Interrupted and pure expansions may have selective topographic toxicity involving preferentially the subcortical or the neocortical structures, causing either isolated parkinsonism or cortical syndromes. Distinct composition and/or localization of CAAs within interrupted expansions could, in turn, be associated with different patterns of neurodegeneration. Finally, the phenotype might be directly impacted by the modification of RNA secondary structure of *ATXN2* transcripts including one or more CAA. CAA motif interruptions decrease the stability of CAG hairpin as indicated by higher ΔG in predicted secondary structure (Additional file 1: Figure S2). These differences can influence the set of RNA-binding proteins interacting with *ATXN2* RNAs, and possibly interfere with RNA processing, localization or translation in specific brain structures [11].

Our patient’s peculiar phenotype could be related to the 27 intermediate allele, which composition is similar that of ALS patients carrying 27 CAG alleles [12]. However he did not have ALS symptoms, and we could not evidence selective loss of Purkinje cells in cerebellar vermis [13], motor neurons alterations, nor cytoplasmic filamentous pTDP-43 inclusions in motor cortex and brainstem characteristic of ALS with intermediate alleles [13, 14]. As such, our patient’s phenotype is more likely related to the 39 repeat expansion.

Finally, our patient showed a rather unique lesional pattern, characterized by FTLD-TDP type A, distinct both from pure or interrupted expansions and intermediate alleles, that expands neuropathological hallmarks associated with *ATXN2* expansions (Additional file 1: Table S3). TDP-43 inclusions were described in one patient carrying 42 pure *ATXN2* expansion carrier [15], as well as in SCA3, SCA7 and Huntington’s diseases, three other CAG expansion disorders. Together, these studies and ours provide robust arguments that common TDP-43 related pathways can be involved, not only in FTLD and ALS, but also in several CAG expansion disorders including interrupted *ATXN2* expansion disease. Based on the present study, it is difficult to assert how interrupted or pure expansions modify the cellular localization of TDP-43 in neurons. However, it has been evidenced that ATXN2 protein ortholog associates with TDP-43, induces its mislocalization and modifies its toxicity in yeast and drosophila models [4].

In summary, this case sheds new light on the significance of *ATXN2* in the spectrum of FTLD and TDP-43 pathologies and raises new challenges in the strategy that has to be applied to reach the molecular diagnosis of FTLD. It enlarges the mutation spectrum of isolated FTLD, showing that *ATXN2* should be analyzed in FTLD patients, or more largely in TDP-43 cases without known FTLD mutations, even in absence of personal or familial history of cerebellar ataxia, ALS or parkinsonism.

## Additional file

Additional file 1: Supplementary methods, cohorts description, molecular analyses and immunostaining. (DOCX 17641 kb)

## Abbreviations

ALS: Amyotrophic Lateral Sclerosis
ATXN2: Ataxin-2
CBS: CorticoBasal Syndrome
CT scan: Computed Tomography scan
FTLD: FrontoTemporal Lobar Degeneration
MRI: Magnetic Resonance Imaging
PolyQ: Polyglutamine
RNA: RiboNucleic Acid
SCA2: SpinoCerebellar Ataxia type 2
SCA3: SpinoCerebellar Ataxia type 3
SCA7: SpinoCerebellar Ataxia type 7
SPECT: Single Photon Emission Computed Tomography
TDP-43: TAR DNA binding Protein 43
pTDP-43: Phospho-TAR DNA binding Protein 43
