# Supplementary material for: Interrupted CAG expansions in ATXN2 gene expand the genetic spectrum of frontotemporal dementias
Source: Acta Neuropathol Commun. 2018 May 30;6:41. doi: 10.1186/s40478-018-0547-8 (PMC5977499; doi:10.1186/s40478-018-0547-8)
Supplement: Supplementary file 1 — Supplementary methods, cohorts description, molecular analyses and immunostaining. (DOCX 17641 kb) [file 40478_2018_547_MOESM1_ESM.docx]

**Supplementary data**

**Interrupted CAG expansions in *ATXN2* gene expand the genetic spectrum of frontotemporal dementias**

Fournier Clémence, PhD^1^*; Anquetil Vincent, PhD^1^*; Camuzat Agnès, MSc^1,2^; Stirati-Buron Sandrine, MD^3^; Sazdovitch Véronique, MD, PhD^5^; Molina-Porcel Laura, MD, PhD^4^; Turbant Sabrina, PhD^1,5^; Rinaldi Daisy, PhD^1,6^; Sánchez-Valle Raquel, MD, PhD^4,7^; Barbier Mathieu, PhD^1^; Latouche Morwena, PhD^1,2^; Neuro-CEB Neuropathology Network**; Stevanin Giovanni, PhD^1,2,8^; Seilhean Danielle, MD, PhD^1,5^; Brice Alexis, MD^1,8^; Duyckaerts Charles, MD, PhD^1,5^; Le Ber Isabelle, MD, PhD^1,6^.

***Supplementary methods***

*Patients’ cohort and molecular analyses*

We studied post-mortem frozen brain tissues from 31 FTLD patients with TDP-43-immunoreactive inclusions (FTLD-TDP), without known related mutation. The samples were collected through the Neuro-CEB brain-biobank (n=10) and the Barcelona-Biobanc-HC-IDIBAPS network (n=21). The cohort is described in supplementary Table 1. The most frequent genetic causes of FTLD, ALS and parkinsonism were excluded in all the cases before their inclusion, either by exome sequencing (*PGRN, MAPT, TARDBP, TBK1, FUS/TLS, VCP, TREM2, CHMP2B, UBQLN2, SQSTM1, MATR3, OPTN, ANG, CSF1R, CHCHD10, PSEN1, PFN1, SOD1, CCNF, DPP6, NEK1, LRRK2, PINK1, SCNA, PARK2* genes), or by repeat-primed PCR (*C9ORF72*).

*Post-mortem* sampling protocols and diagnoses were done as described (Mackenzie *et al.*, 2011). The main clinical diagnosis was bvFTD (n=22), svPPA (n=7) and CBS (n=2), based on international clinical criteria. The clinical and pathological characteristics of the cohort are summarized in supplementary Table 1. The informed consent for *post-mortem* and genetic studies were signed by the patients or by their legal representatives in their name, as allowed by the French and Spanish laws and approved by local ethics committees.

*Molecular analyses.* The *ATXN2* CAG/CAA repeat was PCR amplified on DNA of frontal cortex in all cases. Briefly, 1µM of SCA2-A (5’-GGGCCCCTCACCATGTCG-3’) and SCA2-B (5’-CGGGCTTGCGGACATTGG-3’) primers were used to PCR amplify 20 ng of genomic DNA with TaqDNA polymerase (Qiagen) according to the manufacturer’s protocol. After an initial denaturation at 95°C for 5 min, 35 cycles were repeated with denaturation at 96°C for 1 min, annealing temperature of 63°C for 30 s, extension at 72°C for 1.5 min and a final extension of 5 min at 72°C. CAG repeat allele peaks were determined as previously described, using the number of 33 repeats as the threshold of abnormal expansion (Cancel *et al.*, 1997).

The size was then determined on DNA extracted from other clinically relevant (striatum, mesencephalon) and spared regions (occipital cortex, cerebellum) in one case (patient 5) who carried a repeat expansion in the frontal cortex. DNA from other tissues was not available.

We also determined the CAG/CAA allele composition in patient 5. The expanded and normal alleles were gel purified and cloned into the pGemT-easy plasmid (Promega). The insert was sanger-sequenced using T7 and SP6 primers.

*Modeling of ATXN2 RNA secondary structure.* The RNA secondary structures and stability of both alleles of patient 5 were predicted using the ViennaRNA Package (<http://www.tbi.univie.ac.at/RNA>), and compared to an interrupted 37 CAG allele carried by another bvFTD-ALS patient (Bäumer *et al.*, 2014), to pure CAG expansion of the same length, and to the most common 22 and 23 CAG normal alleles (Lorenz *et al.*, 2011). RNA stability was estimated by minimum free energy prediction (ΔG), computed using default parameters; a lower ΔG being predictive of RNA with higher stability.

*Neuropathology*

Middle frontal gyrus, primary sensory motor, primary visual cortices, supramarginal and superior temporal gyri, hippocampus, lenticular nucleus, thalamus, mesencephalon including *substantia nigra*, pons including *locus coeruleus*, medulla oblongata and cerebellum were analyzed. Immunohistochemistry studies were performed with the Ventana BenchMark XT strainers using antibodies against the following antigens: TDP-43 (PTG; polyclonal; 1/2000), phosphorylated TDP-43 (pTDP-43) (Cosmo Bio, pS409/410-2, 1/5000), polyglutamine repeats (1C2, Eurogenex; 1/4000), ubiquitin (Dako, Poly, 1/500), p62 (3/p62 LCK, Biosciences, 1/500), neurofilament (Dako, 2F11, 1/2000), Abeta (Dako; 6F/3D, 1/200), tau (Innogenetics, AT8; 1/500) and alpha-synuclein (Zymed, LB 509, 1/250), as described (Seilhean *et al.* 2011). The harmonized neuropathological classification (Mackenzie *et al.*, 2011) was applied.

*ATXN2 immunostaining.* Histological examination was performed on 5µm section cut from formalin-fixed paraffin embedded tissues. Paraffin-embedded sections were first deparaffinized with xylene and ethanol. After brief wash with deionized water, antigen retrieval was performed using decloaking chamber 3 min in 100 mM citrate buffer (pH 6.0). Sections were allowed to settle for 10 min followed by a brief wash with deionized water. Endogenous peroxidase was blocked with 0.3% H2O2 in 40% methanol for 5 min. After rinse with deionized water, blocking was performed with 4% BSA in PBS-0.2% Triton X100 for 1hour. Incubation with anti-Ataxin-2 (BD Biosciences, 1/500) was performed over-night at 4°C in blocking solution. Biotinylated horse anti-mouse secondary antibody (Vector Laboratories) was additionally used at 1:250 dilution in blocking solution for 1h at room temperature. Sections were developed with a standard ABC Elite kit (Vector Laboratories) using DAB (3-3’ diaminobenzidene) as chromagen, then counterstained with Mayer’s Hematoxylin. Images were obtained with DFC295 (Leica) with 20x, 40x or 63x objectives.

***Supplementary results***

*Molecular analyses.*

An *ATXN2* pathogenic full-length CAG repeat was detected in one (patient 5) of the 31 patients (3%). He carried a 39 CAG expansion, and a 27 CAG intermediate allele (Supplementary Fig. 1). The 39 CAG expanded allele was interrupted by four CAA motifs: CAG_8_–CAA–CAG_4_–CAA–CAG_4_-CAA-CAG_9_-CAA-CAG_10_. The 27 CAG allele included three CAA motifs: CAG_8_–CAA–CAG_4_–CAA–CAG_4_-CAA-CAG_8_. The 39 CAG/CAA expansion and the intermediate 27 repeats were identified in all brain structures studied (frontal, occipital cortices, striatum, mesencephalon, cerebellum).

*Predicted secondary structures of human ATXN2 transcripts with repeat expansions.*

CAA motifs are located at the external hairpin loop or in symmetrical internal loop within interrupted expansions and in the normal alleles, whereas intermediate or pure expanded alleles containing 27, 37 or 39 CAG form single hairpins. Pure 27, 37 and 39 CAG repeat expansions are all more stable (lower ΔG, Supplementary figure 2B) than interrupted ones, indicating greater stability of uninterrupted than interrupted RNA structures.

*Clinical description of the patient carrying interrupted 39 CAG repeat expansion (patient 5, see supplementary table 1).*

The patient (patient 5) carrying interrupted 39 CAG repeat expansion developed progressive agrammatism, word omissions and dysarthria, suggestive of nfvPPA, at age 70. The mini-mental status (MMSE) was scored 23/30 at age 72 (attention: 1/5; language: 6/8). At age 73, marked planning, attention, inhibition, mental flexibility deficits and perseverations were noted. The MMSE was 11/29 with language (2/7), spatial orientation (1/5), attention (0/5), praxis (0/1) and verbal memory recall (0/3) deficits. Bilateral, predominantly right, ideomotor apraxia (praxis score: 4/23) and visuo-constructive deficits were present. Confrontation naming, visual gnosis and visual recognition memory were spared. Immediate (94%) and delayed recall (94%) of the DMS-48 were normal. Examination showed bilateral grasping, rigidity and akinesia, but no cerebellar syndrome. Brain imaging revealed frontal and predominantly left peri-sylvian and parietal atrophy. Cerebellum, brainstem and hippocampus were normal (Fig. 1a, b, c). Brain HMPAO-SPECT showed predominantly left prefrontal and parietal hypoperfusion (Fig. 1d). A CBS was diagnosed, based on clinical and neuroimaging criteria (Armstrong *et al.*, 2013). The patient died at age 77. A post-mortem examination was performed. The patient had a single child; no information was available about the parents and child.

*Post-mortem examination of the brain.*

The brain weighted 1,330 g. Macroscopic examination revealed marked frontal and temporal atrophy, atrophy of the Ammon’s horn (CA1) and of the subiculum. Neuronal loss and gliosis were severe in the middle frontal gyrus, motor cortex and supramarginal gyrus. It mainly involved the superficial layers and were associated with a superficial laminar spongiosis. The superior temporal gyrus and the visual cortex were normal. There was a severe neuronal loss with gliosis in CA1 sector of the hippocampus and in the subiculum (hippocampal sclerosis). The *substantia nigra* was depigmented. The pons (including the *locus coeruleus*) was normal. Numerous axonal spheroids, neurofilament-positives, were found in the amiculum of the inferior olive. The cerebellum and the dentate nucleus were normal (Fig. 1e). TDP-43, pTDP-43, p62 and ubiquitin immunohistochemistry revealed small round cytoplasmic inclusions, sometimes glial, more abundant in the superficial layers of the middle frontal gyrus, motor cortex, and supramarginal gyrus (Fig. 1f, g, h). Rare pTDP-43 ‘cat eye’ intranuclear inclusions were detected (Fig. 1i). A few cytoplasmic inclusions were found in the dentate gyrus. Scarce TDP-43 and pTDP43 positive neurites were present, mainly in the frontal cortex and the supramarginal gyrus (Fig. 1g). There was no TDP-43 inclusions in the brainstem. No skein like inclusions were observed in the hypoglossal nucleus. The presence of TDP-43 positive cytoplasmic inclusions, mainly distributed in the upper layers of the cortex, lead to the diagnosis of type A FTLD-TDP (Mackenzie *et al.*, 2011) Ubiquitin and p62 immunohistochemistry did not reveal inclusions in the cerebellum. No intranuclear inclusions were detected with 1C2 antibodies. No alpha-synuclein immunostaining was noted in the *substantia nigra*. Diffuse and focal Aβ deposits were found in the isocortex; they were rare in the hippocampus and the basal ganglia (Thal amyloid phase III). The wall of some middle-sized and large vessels were Aβ positive in the neocortex and the cerebellum (cerebral amyloid angiopathy Thal type 2). AT8 positive neurofibrillary tangles and neurites were found in the transentorhinal and entorhinal areas (Braak stage II) but not is the supramarginal gyrus. This findings correspond to a low score for Alzheimer disease changes (Montine *et al.*, 2012).

*ATXN2 immunostaining.* The effect of the interrupted expansion on Ataxin 2 localization in different brain regions was evaluated in our patient (patient 5) and compared to a control, an ataxic SCA2 patient and an ALS patient with 32 CAG intermediate allele. Examination of ataxin 2 expression and localization in cerebellum and spinal cord of our case revealed a granular cytoplasmic labelling in Purkinje cells for cerebellum and in spinal cord neurons (Supplementary Fig. 3). In frontal cortex, weak diffuse cytoplasmic staining was observed in some neurons. In the frontal cortex and spinal cord of control, Ataxin 2 was localized throughout the cytoplasm in a diffuse pattern, whereas no Ataxin 2 staining was observed in the cerebellum (Supplementary Fig. 3). The same localization of Ataxin2 in the spinal cord was observed in the SCA2 patient, but no labelling of any cell types could be evidenced in frontal cortex and cerebellum (Supplementary Fig. 3). The specific granular staining in the Purkinje cells of our case was also found in an ALS case with intermediate 32 CAG expansion (Supplementary Fig. 3).

***Supplementary references***

Cancel G, Dürr A, Didierjean O, Imbert G, Bürk K, Lezin A, et al. Molecular and clinical correlations in spinocerebellar ataxia 2: a study of 32 families. Hum. Mol. Genet. 1997; 6: 709–15.

Lorenz R, Bernhart SH, Höner Zu Siederdissen C, Tafer H, Flamm C, Stadler PF, et al. ViennaRNA Package 2.0. Algorithms Mol. Biol. AMB 2011; 6: 26.

Seilhean D, Le Ber I, Sarazin M, Lacomblez L, Millecamps S, Salachas F, et al. Fronto-temporal lobar degeneration: neuropathology in 60 cases. J. Neural Transm. Vienna Austria 1996 2011; 118: 753–64.

Montine TJ, Phelps CH, Beach TG, Bigio EH, Cairns NJ, Dickson DW, et al. National Institute on Aging-Alzheimer’s Association guidelines for the neuropathologic assessment of Alzheimer’s disease: a practical approach. Acta Neuropathol. (Berl.) 2012; 123: 1–11.

***Supplementary figures***


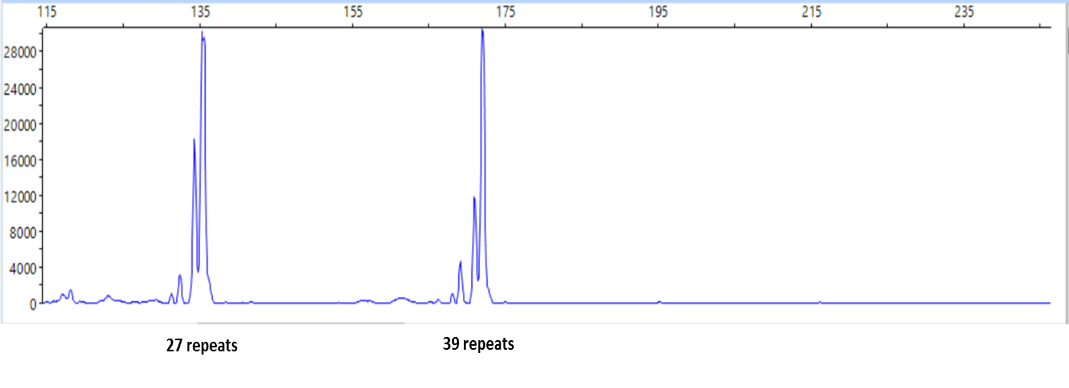


**Supplementary Figure 1.** **Fragment analysis from an automated ABI3730 sequencer of PCR amplified DNA extracted from frontal cortex of patient 5.** Two peaks of 27 and 39 repeats were identified. The number of repeats carried by each allele is written below the corresponding peak.

○●○○○●○○○○●○○ 27-31 CAG/CAA: ALS risk

○●○○○●○○○ 13-26 CAG/CAA: Normal

○○○○○○○○○○○○○○○○○○ >34 CAG: SCA2

○●○○○●○○○○●○○○○●○ 34-39 CAG/CAA: Parkinson disease & FTLD

A.

B.


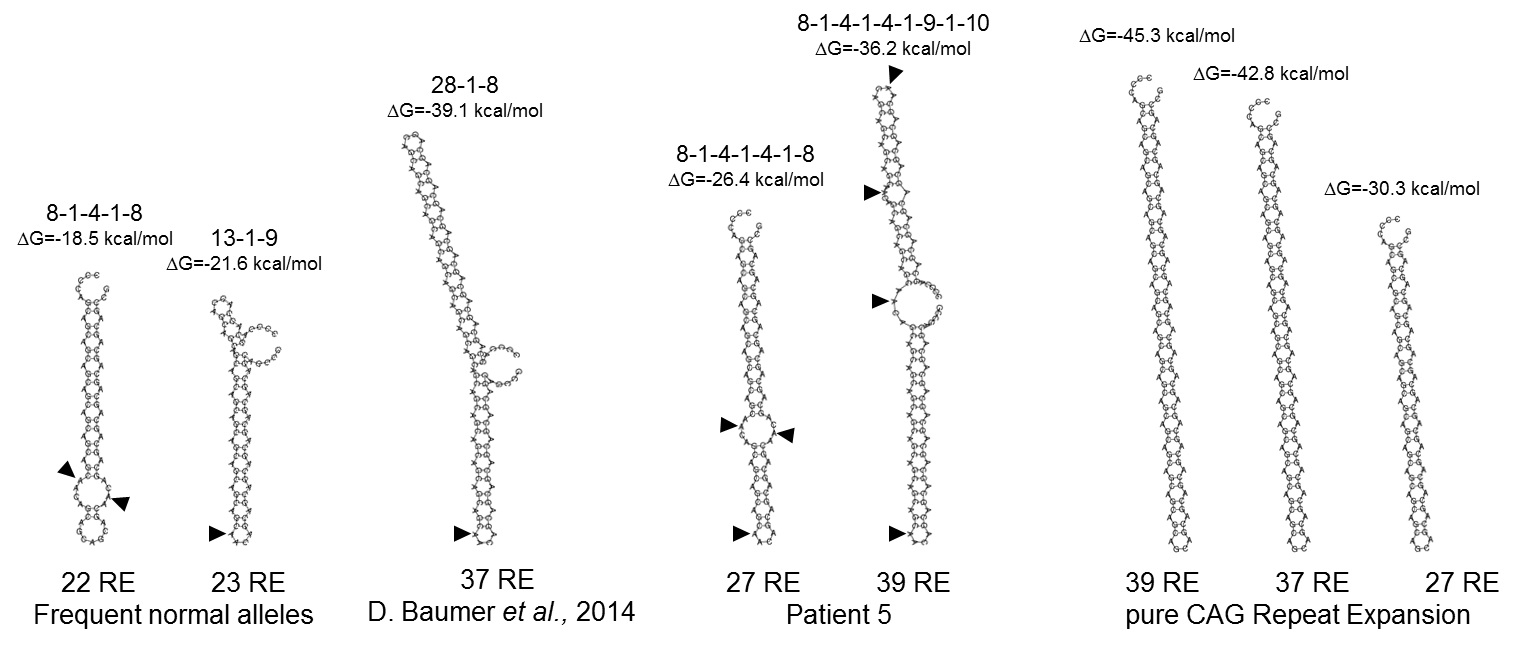


**Supplementary figure 2. *ATXN2* CAG and CAG/CAA repeat sequences in different associated phenotypes (A), and predicted secondary structures of several repeat expansions in human *ATXN2* transcripts (B).** Modelisation of RNA secondary structures of the most frequent normal alleles (22 and 23 CAG repeats), of an interrupted 37 CAG repeat allele carried by a FTLD-ALS case (Baümer et al., 2014), of both CAG repeat alleles (27 and interrupted 39 CAG) carried by patient 5, as well as the corresponding length of uninterrupted structures. Interrupted intermediate-size and expanded alleles, as well as the normal repeat alleles have CAA (arrows) located at the external hairpin loop and/or in symmetrical internal loop. Mutant alleles of FTLD cases have longer and more stable hairpin structure than the most frequent normal alleles. The uninterrupted pure CAG repeats present long tracts with more stable hairpin than interrupted expansions. RE: repeat expansions.


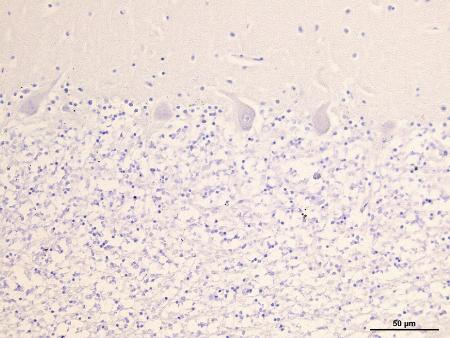


b


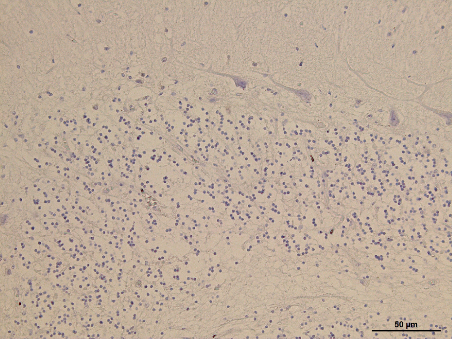


c


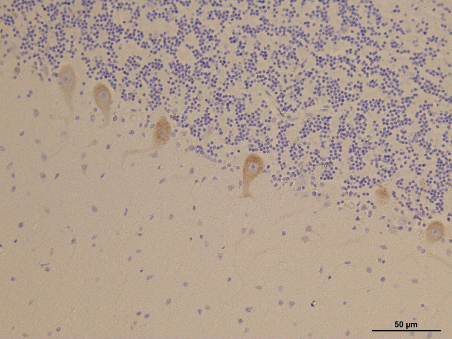


a


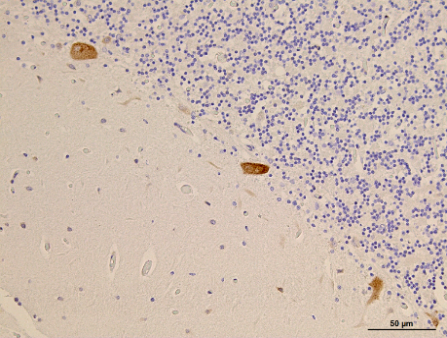


d


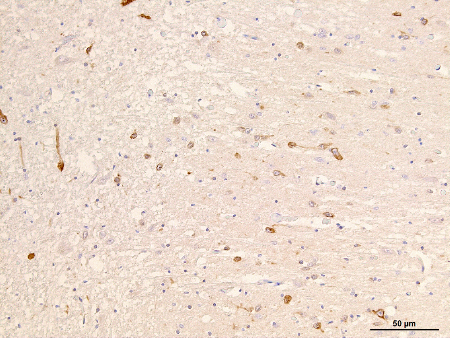

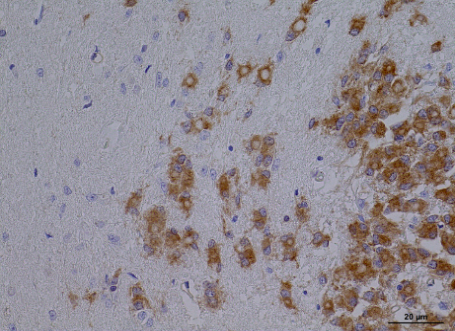

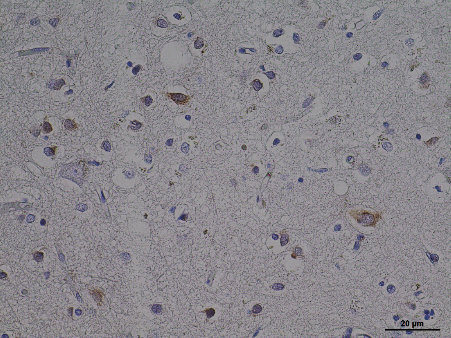

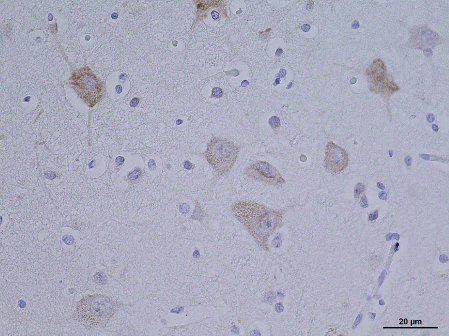

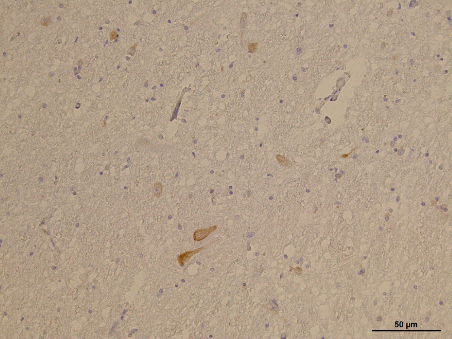

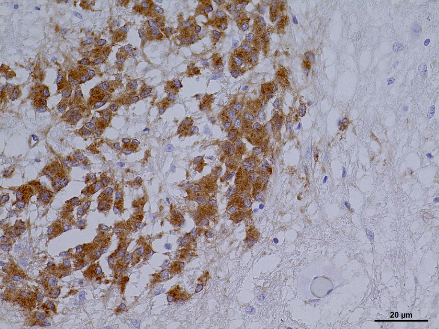


i

m

g

j


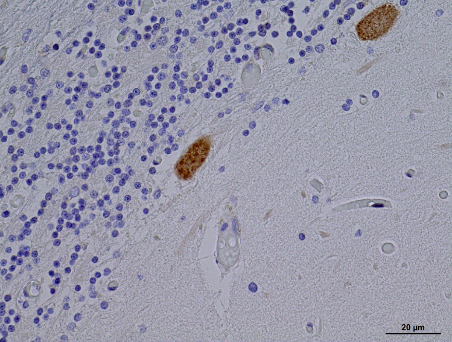

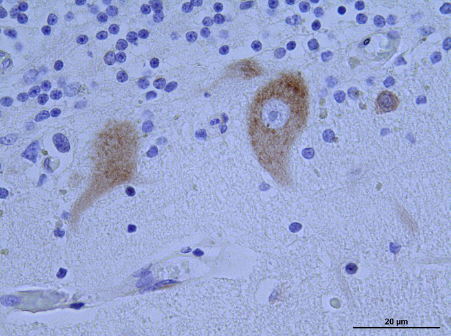


e

f


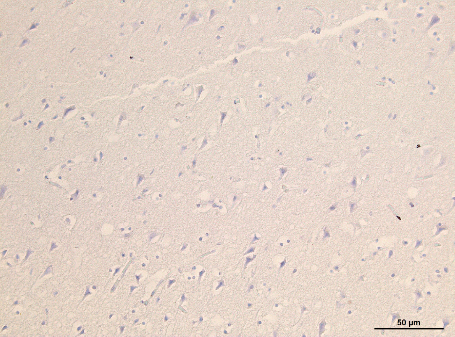


h


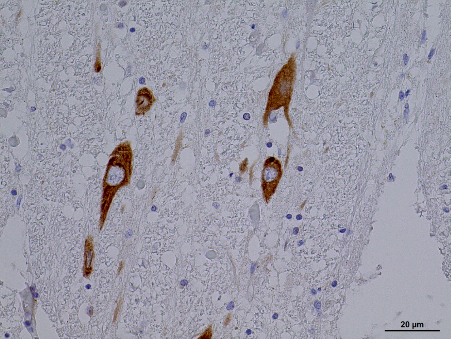


k

l


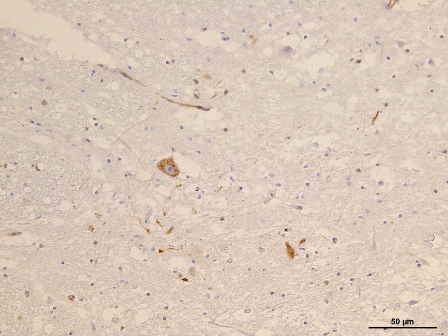


n

o

**Supplementary Figure 3**. Ataxin-2 immunostaining. a-f. Cerebellum. Positive granular cytoplasmic immunostaining of Purkinje cells in our case (Patient 5), 20x (a); Negative immunostaining in healthy control, 20x (b); Purkinje cell loss but no Ataxin-2 immunostaining in a SCA2 patient carrying uninterrupted *ATXN2* expansion, 20x (c); Positive granular cytoplasmic immunostaining in an ALS patient carrying intermediate allele of 32 CAG repeats, 20x (d), 40x (e), 63x (f). g-i. Frontal cortex. Weak diffuse cytoplasmic immunostaining in some neurons in patient 5, 40x (g); in healthy control, 40x (h); in SCA2 patient carrying uninterrupted *ATXN2* mutation, 40x (i). j-o: Spinal cord. Positive granular cytoplasmic immunostaining in patient 5, 20x (j), 40x (k); in a healthy control, 20x (l), 40x (m); in a SCA2 patient carrying uninterrupted *ATXN2* mutation, 20x (n), 40x (o). Scale bars: 20 or 50µm.

| **ID samples** | **Clinical diagnosis** | **Age at onset, ys** | **Age at death, ys** | **Pathological FTLD-subtype** |
| --- | --- | --- | --- | --- |
| 1 | bvFTD | 74 | 84 | FTLD TDP type A |
| 2 | svPPA | 64 | 77 | FTLD TDP type C |
| 3 | svPPA | 57 | 68 | FTLD TDP type C |
| 4 | bvFTD | 66 | 77 | FTLD TDP type B |
| **5** | **CBS** | **70** | **77** | **FTLD TDP type A** |
| 6 | svPPA | 58 | 72 | FTLD TDP type B |
| 7 | bvFTD | 57 | 61 | FTLD TDP type B |
| 8 | bvFTD | 58 | 69 | FTLD TDP |
| 9 | bvFTD | 64 | 77 | FTLD TDP type B |
| 10 | bvFTD/PARK | 68 | 84 | FTLD TDP type A |
| 11 | svPPA | 66 | 78 | FTLD TDP type C |
| 12 | bvFTD | 77 | 88 | FTLD TDP type A |
| 13 | bvFTD | 65 | 85 | FTLD TDP type C |
| 14 | bvFTD | 50 | 56 | FTLD TDP type A |
| 15 | bvFTD | 50 | 62 | FTLD TDP type C |
| 16 | bvFTD | na | 77 | FTLD TDP type B |
| 17 | bvFTD | 59 | 70 | FTLD TDP type C |
| 18 | bvFTD | 78* | 83 | FTLD TDP type B |
| 19 | bvFTD | 72 | 80 | FTLD TDP type A |
| 20 | svPPA | 64 | 77 | FTLD TDP type C |
| 21 | svPPA | 52 | 62 | FTLD TDP type C |
| 22 | bvFTD | 83 | 84 | FTLD TDP type C |
| 23 | bvFTD | 68 | 76 | FTLD TDP type A |
| 24 | bvFTD | 73* | 82 | FTLD TDP type A |
| 25 | bvFTD | 68 | 71 | FTLD TDP type A |
| 26 | bvFTD | 63 | 70 | FTLD TDP type C |
| 27 | bvFTD | 74* | 77 | FTLD TDP type C |
| 28 | bvFTD | 81 | 90 | FTLD TDP type A |
| 29 | svPPA | 59 | 74 | FTLD TDP type C |
| 30 | CBS | 77 | 84 | FTLD TDP type A |
| 31 | bvFTD | 82* | 86 | FTLD TDP type B |

**Supplementary table 1. Summary of clinical diagnosis and pathological characteristics of 31 FTLD-TDP-43 cases included in the study.** bvFTD: behavioral variant of FrontoTemporal Dementia, FTLD: FrontoTemporal Lobar Degeneration, svPPA: semantic variant of Primary Progressive Aphasia, TDP: TAR DNA Binding Protein, CBS: CorticoBasal Syndrome, PARK: Parkinsonism , ys: years, *= age at diagnosis. The patient (patient 5) described in the paper, carrying a 39 CAG *ATXN2* interrupted repeat expansion, is indicated in bold.

|  | **TDP-43 staining** | **1C2 staining** | **ATXN2 staining** | **α-SYN staining** |
| --- | --- | --- | --- | --- |
| Full-length uninterrupted expansion, SCA2 phenotype,  2 cases  *(Estrada et al., 1999; Toyoshima et al., 2011)* | + Mostly NCI  Few ‘cat eyes’ NII  (1 case) | + Mostly NCI, rare NII | NA | NA |
| Full-length interrupted expansions with parkinsonism phenotypes, 2 cases  *(Takao et al., 2011; Yomono et al., 2010)* | NA | + | NA | + |
| Intermediate alleles with ALS phenotype,  10 cases  *(Hart et al., 2012; Highley et al., 2016)* | + Skein-like/filamentous NCI | - | +/- (variable, in 4 cases) | NA |
| Full-length interrupted expansion, FTLD-ALS phenotype, 1 case *(Bäumer et al., 2014)* | + Mostly NCI  Few ‘cat eyes’ NII | + | + | NA |
| Full-length interrupted expansion, FTLD phenotype, 1 case *(This study)* | + Mostly NCI  Few ‘cat eyes’ NII | - | + | - |

**Supplementary table 2. Overview of the pathological hallmarks characterizing *ATXN2* uninterrupted and interrupted expansions, as well as intermediate alleles.** TDP-43: TAR DNA Binding Protein 43, ATXN2: Ataxin 2, α-SYN: alpha-Synuclein, NCI: Neuronal Cytoplasmic Inclusion, NII: Neuronal Intranuclear Inclusion. NA: not available
